# A Case of Infantile Reversible Cytochrome C Oxidase Deficiency Myopathy in Taiwan: A 4-Year Follow-Up

**DOI:** 10.1155/carm/1823517

**Published:** 2025-02-18

**Authors:** Yu-Ting Ma, Ju-Li Lin, Ming-Wei Lai, I-Jun Chou, Mao-Sheng Hwang

**Affiliations:** ^1^Department of Pediatrics, Division of Genetics and Pediatric Endocrinology, Linkou Chang Gung Memorial Hospital, Taoyuan, Taiwan; ^2^Department of Pediatrics, Division of Pediatric Gastroenterology, Linkou Chang Gung Memorial Hospital, Taoyuan, Taiwan; ^3^Liver Research Center, Linkou Chang Gung Memorial Hospital, Taoyuan, Taiwan; ^4^Chang Gung University College of Medicine, Taoyuan, Taiwan; ^5^Department of Pediatrics, Division of Pediatric Neurology, Chang Gung Children's Hospital at Linkou, Taoyuan, Taiwan; ^6^Department of Pediatrics, Chang Gung Memorial Hospital, Taoyuan, Taiwan

**Keywords:** benign COX deficiency myopathy, cytochrome C oxidase, mitochondria

## Abstract

Infantile reversible cytochrome c oxidase (COX) deficiency myopathy is a mitochondrial rare disease with onset age of first day to three months with symptoms of generalized muscle weakness and severe hypotonia. Despite its initial serious conditions, the symptoms may improve spontaneously later in their life, with the so-called “benign” myopathy accordingly. This benign mitochondrial myopathy might be improved in their later life, which is different from most mitochondrial myopathies with progression by age. Therefore, we depicted the rare case of her clinical course during our medical practice, anticipating to provide more information of this rare disease.

## 1. Introduction

Infantile reversible cytochrome c oxidase (COX) deficiency myopathy, which is also recognized as benign COX deficiency myopathy, is a mitochondrial rare disease with onset age of first day to three months [1, 2]. Their symptoms were generalized muscle weakness and severe hypotonia [1, 2]. Despite its initial serious conditions, the symptoms might improve spontaneously between 4 and 36 months and could be back to normal by 2 or 3 years or with only mild myopathy left in adulthood [1–4].

COX, the same as respiratory chain complex IV, is an enzyme comprised of 13 subunits [5]. Mitochondrial DNA (mtDNA) encoded three of the subunits and nuclear DNA encoded the other 10 subunits [5]. Because of the difference of the enzyme, COX deficiency myopathy can be divided into two categories as following: one dominated by muscle involvement disease such as fatal infantile myopathy and benign infantile myopathy; the other was related to brain disease such as Menkes' disease, myoclonic epilepsy with ragged red fibers (MERRFs), Alpers syndrome, and Leigh syndrome [5].

In spite of the complexity of COX, in benign COX deficiency myopathy, the genetic cause is by mtDNA homoplasmic 14674T > C missence mutation in the MTTE gene [1]. On the other hand, fatal COX deficiency myopathy is inherited in autosomal recessive pattern such as SCO_2_ gene and SURF1 gene homozygous or compound heterozygous mutations. Genetic test should be performed initially under the suspicion of COX deficiency myopathy because clinical features in infancy between fatal type myopathies and benign type myopathies are indistinguishable at first, but with dramatic different prognosis in their entire life [1].

Here, we presented one case of benign COX deficiency myopathy in Linkou Chang Gung Memorial Hospital.

## 2. Case Presentation

This 2-month-old female was admitted to our pediatric ward due to poor appetite for 2 weeks. She was born Gravida 2 Para 2 (G2P2) under gestational age 40 weeks with birth body weight 3200g (15^th^–50^th^ percentile) via normal spontaneous delivery. Birth body height and birth head circumference were undocumented. She was generally healthy after birth without systemic diseases before this event 2 weeks ago and she had received first dose of hepatitis B vaccine as scheduled.

When admitted, she was found failure to thrive with body weight: 3.8 kg (< 3^rd^ percentile), body length: 54 cm (3^rd^–15^th^ percentile), and head circumference: 38 cm (15^th^–50^th^ percentile). Feeding amount of the patient decreased from regular formula S26 100–120 mL per 3-4 h to 30–50 mL per 3-4 h. Accompanying symptoms included distended abdomen and poor activity. She had solid stool passage every day with yellowish to greenish color without blood tinged nor mucus in stool. There was no fever, vomiting, diarrhea, skin lesion, or other respiratory symptoms.

Before admission into our ward, she was treated in another hospital for 1 week. Treatment included hydration and changing her diet to partial hydrolyzed formula after basic workup. Her appetite improved after treatment and the patient was discharged accordingly. However, 5 days later after discharge, decreased urine output with poor appetite and poor activity recurred. She was brought to our emergency department by the decision of parents.

On physical examination, distended abdomen with normoactive bowel sound and tachyarrhythmia (heart rate: 165 beats per minute) were found. Abdominal x-ray and abdominal ultrasound revealed flatulence without obstruction; EKG revealed sinus tachycardia; laboratory exam revealed mild leukocytosis white blood cell (WBC): 12900/μL (normal range: 5300–12000/μL at her age) and thrombocytosis (platelet: 720k/uL, normal range: 150–400 k/μL at her age); elevated aspartate transaminase (AST): 111 U/L (9–80 U/L); and alanine transaminase (ALT): 63 U/L (13–45 U/L). The level of C-reactive protein (CRP), electrolyte, and troponin I were all within normal range.

After admission, more detailed exams were done. Neurologic examination showed significant hypotonia, muscle weakness with absence of deep tendon reflex and negative Babinski sign. On admission day 2, we inserted nasogastric tube (NG tube) for feeding because of unresolved poor appetite and feeding difficulty due to muscle weakness. Further laboratory test showed elevated AST: 159 U/L, ALT: 67 U/L, ammonia: 116 μg/dL (< 94 μg/dL), lactate: 65 mg/dL (4.5–19.8 mg/dL), creatinine kinase (CK): 738 U/L (20–180 U/L), and metabolic acidosis (vein blood gas: pH: 7.296, pCO2: 40.3 mmHg, pO_2_: 41.6 mmHg, cHCO3: 19.2 mmol/L, BEecf: −7.3 mmol/L). We concluded that this patient had significant progressive peripheral hypotonia which resulted from muscle damage that accompanied with elevated liver enzymes. Serum Cu and ceruloplasmin were arranged to rule out Menkes disease with symptoms of light hair color, hypotonia, and feeding difficulty, and the results revealed only mild decreased Cu level: 79.4 μg/dL (80–153 μg/dL). As an X-linked recessive disease, Menkes disease can also be ruled out by the absence of Menkes disease symptoms by female patient's father. Protein intake was adjusted from 2.2 to 1.5 g/kg/day for elevated ammonia. Otherwise, ketone, zinc, and spinal muscular atrophy screening tests were all normal. Brain echo revealed bilateral subependymal cyst (left size: 6.5∗5.0 mm and right size: 8.7∗2.5 mm). Cardiac echo revealed borderline interventricular septal hypertrophy and patent foramen ovale: 1.2 mm (left to right shunt).

For elevated CK with borderline interventricular septal hypertrophy and lactic acidosis, we arranged mitochondrial DNA screening test for suspicious mitochondrial myopathy. The result of the screening showed homoplasmic 14,674 T > C point mutation in mitochondrial MT-TE gene, which is a confirmed pathogenic mutation of infantile reversible COX deficiency myopathy. We, therefore, added ubidecarenone 10 mg/tab 1/2 # BID and vitamin B6 50 mg/tab 1/2 # QD for the female infant. Her appetite gradually improved to 90 mL per 4 h after our treatment before discharge. Regular laboratory exam checkup and dose adjustment of drugs at outpatient department were mentioned as below (Table 1). The CK level was monitored one to two months at first and then regularly followed up every 6 months. We had tried stopping her ubidecarenone and vitamin B6 when she was 2 years and 7 months old when CK and lactate level were both normal for 17 months. However, elevated CK level (344 U/L) was found when she was 4 year old after drug cessation for 17 months during our regular follow up. Therefore, we had added back ubidecarenone and vitamin B6 so far with the improvement of CK level thereafter.

We also arranged regular cardiac echo every 6 months for borderline interventricular septal hypertrophy follow-up. Her cardiac structure returned to normal since 1 year and 2 months old. However, it deteriorated to slightly hypertrophic left ventricle at 4 years old.

The patient removed her NG tube after 6 months use by herself. Her body weight increased to 8.2 kg (50^th^–75^th^ percentile) at that time. NG tube was not inserted again consequently. Her body weight increased steadily thereafter.

Owing to the inheritance of mitochondrial disease, we recorded more family histories of the maternal side. The mother was mentioned hypotonia before one year old. One brother of the mother and the grandmother had no similar symptoms. The patient had one older brother. He had clinical symptom of leg pains easily triggered by strenuous activity but gradually improved when grew up. We speculated that the mother and the older brother may also carry this mitochondrial point mutation despite the lack of genetic evidence, which should be investigated in the future.

## 3. Discussion

In our patient, the mild myopathy recurred when 4 years old after drug cessation for 17 months. In previous reports, myopathy might spontaneously return to normal by the age of 2 or 3 years old [1–3]. One case was found free of clinical symptoms when 4 years old [3]. However, there were still some patients with mild myopathy in their adulthood [1]. The CK level of our patient was improved after taking ubidecarenone again when 4 years and 7 months old. We might try to discontinue her medications when her CK level remains within normal range for extended periods, according to the good prognosis of this disease.

The cardiac ultrasound showed normal chamber size without interventricular septal hypertrophy since 1 year and 2 months old, compatible with the timing of recovery of CK and lactate level. During serial follow-up, slightly hypertrophic left ventricle was found when 4 years old. It was also compatible with the timing of disease progression in this patient. The possible progression time was not recorded based on current literatures since most of patients in this disease became symptoms free or remained only mild myopathy as they became older.

In some patients, liver may be involved with hepatomegaly and macroglossia may be seen [1, 3, 6]. Rita Horvath et al. reported all of their 17 patients without brain, peripheral nerves, and cognitive function dysfunction in the initial state of the disease, but one of the 17 patients developed neurological symptoms in later age [1]. In our patient, there were no abovementioned symptoms so far.

Infantile reversible COX deficiency myopathy may be caused by homoplasmic 14,674T > C or 14,674T > G in mitochondrial MT-TE gene, but the former is more common than the latter. Mimaki et al. reported 8 Japanese patients with 6 patients showing homoplasmic 14,674T > C and the other two patients showing homoplasmic 14,674T > G [2]. In the literature of Rita Horvath et al., 17 White Caucasians patients from 12 families all showed homoplasmic 14,674T > C [1]. J Uusimaa et al. also reported 5 Caucasian patients from 4 families revealed homoplasmic 14,674T > C [7]. In our patient, it presented with homoplasmic 14,674T > C in mitochondrial MT-TE gene, which was the same as most of other patients.

Similar cases were reported in Taiwan before. T.-H. CHEN et al. reported two cases of infantile reversible COX deficiency myopathy with the onset age of 6 weeks old and 14 days old, respectively. M. 14674T > C mutation was confirmed of both patients [8]. The mother of one patient in this case report was also diagnosed with this disease with the same mutation, but patient's mother and the whole maternal family were all lack of clinical symptoms. We thought it may be due to the inattention or mild symptoms in their early lives of the family or it may be due to the heteroplasmy in their muscle tissues, which leads to the possibility of no clinical symptoms.

Until now, the mechanism of spontaneous recovery in this disease is still under investigation, and the incidence of this rare disease is not clearly understood so far. Further clinical cases should be gathered up for more statistical analysis and study in the future.

In conclusion, infantile reversible COX deficiency myopathy is a benign mitochondrial disease mainly caused by mitochondrial MT-TE gene homoplasmic 14,674T > C. Some patients may be free of symptoms by age of 2–4 but some patients may remain mild myopathy in their adulthood. Careful follow-up should be done in every patient persistently for possible subtle muscle damage including regularly physical examination, CK level, and cardiac echography at least every 6 months since recurrent muscle damage may occur after ceasing the medication when symptoms subsided. For some patients who may suffer from hepatomegaly, macroglossia, or neurologic symptoms, regular abdominal echography should also be considered and regular physical exam along with history taking about breathing status, swallowing condition, and cognitive function should also be done.

## Figures and Tables

**Table 1:** Serial laboratory results and the corresponding drug adjustments.

Age	2 months old	3 months old	5 months old	9 months old	1 year and 2 months old	1 year and 10 months old	2 year and 4 months old
CK (U/L)	738↑	273↑	208↑	148	145	129	158
Lactate (mg/dL)	65↑	66.8↑		28.4↑	14.9	14	12.8
Ammonia (μg/dL)	116↑	71	61				
ALT (U/L)	67↑		32			21	
AST (U/L)	159↑	35				41	
Drug adjustment	Add ubidecarenone 10 mg/tab 1/2 # BID and Vitamin B6 50 mg/tab 1/2 # QD		Titrated up ubidecarenone 10 mg/tab to 1 # BID			Tappered down ubidecarenone 10 mg/tab to 1 # QD	

**Age**	**2 year and 7 months old**	**3 years and 1 month old**		**4 years old**		**4 years and 7 months old**	

CK (U/L)	171	179		344↑		249↑	
Lactate (mg/dL)	12.7	15.5		17.3		11.6	
Drug adjustment	Stop ubidecarenone and Vitamin B6			Add back ubidecarenone 10 mg/tab 1 # BID and Vitamin B6 50 mg/tab 1/2 # QD			

## Data Availability

The data that support the findings of this study are available in the supporting material of this article.
